# Neutrophil‐lymphocyte ratio and plasma lactate predict 28‐day mortality in patients with sepsis

**DOI:** 10.1002/jcla.22942

**Published:** 2019-07-02

**Authors:** Yunlong Liu, Jie Zheng, Daisong Zhang, Liling Jing

**Affiliations:** ^1^ Baoshan Branch Shuguang Hospital Affiliated to Shanghai University of Traditional Chinese Medicine Shanghai China; ^2^ Changhai Hospital The Second Military Medical University Shanghai China; ^3^ Department of Surgery Penglai People's Hospital Penglai China

**Keywords:** mortality, neutrophil‐lymphocyte ratio, plasma lactate, prognosis, sepsis

## Abstract

**Objective:**

The predictive potential of the neutrophil‐to‐lymphocyte ratio (NLR) and plasma lactate was investigated in regard to the prognosis of patients with sepsis.

**Methods:**

Sixty‐three nonsurgical and nontrauma adult patients with sepsis admitted to the intensive care unit (ICU) from September 2016 to October 2018 were consecutively included in the study. In addition, healthy subjects were assigned to a control group. Neutrophil and lymphocyte counts were evaluated via a complete blood count. Plasma lactate, procalcitonin (PCT), and C‐reactive protein (CRP) levels were measured. The main outcome was 28‐day mortality.

**Results:**

Neutrophil‐to‐lymphocyte ratio and plasma lactate levels of the patients were significantly higher than those of control subjects: 19.44 (14.3‐34.53) vs 14.09 (8.17‐28.99), *P* = 0.049; and 3.7 (3‐6.6) vs 2.72 (2.13‐4.3) ng/mL, *P* = 0.008, respectively. There were no statistical differences in the concentrations of PCT and CRP between nonsurvivors and survivors: 6.1 (3.43‐33.59) vs 9.43 (4.24‐37.68) ng/mL, *P* = 0.44; and 108 (77.8‐153) vs 114.5 (71.43‐162) ng/mL, *P* = 0.672, respectively. With an optimal cutoff of 14.08, the sensitivity and specificity of NLR for prediction of 28‐day mortality were 78.3% and 50%, respectively. And the sensitivity and specificity of plasma lactate level to predict 28‐day mortality, at an optimal cutoff value of 2.99 mmol/L, were 82.6% and 55%, respectively.

**Conclusions:**

Neutrophil‐to‐lymphocyte ratio and plasma lactate were associated with poor outcomes in patients with sepsis and predicted mortality.

## 1 INTRODUCTION

Sepsis is the primary cause of death from infection. It is also one of the leading causes of intensive care unit (ICU) mortality, despite early administration of antibiotics and hemodynamic management.^1^ Sepsis is a life‐threatening organ dysfunction caused by a deregulated host response to an infection. It is a major public health concern, accounting for more than $20 billion in the US hospital costs in 2011.^2^ Furthermore, survivors of sepsis often suffer from substantial and persistent new cognitive impairment and functional disability, imposing a great burden on healthcare systems and societies worldwide.^3^

Therefore, finding tools that may assist physicians in early identification of patients with sepsis, especially those at high risk, may improve outcomes in patients with sepsis. Plasma biomarkers may have diagnostic, prognostic, and theranostic values, all of which are important factors influencing outcomes in patients with sepsis.^4^ Indeed, procalcitonin (PCT) and C‐reactive protein (CRP) had been applied in evaluation of the severity of an episode of sepsis along with patient therapeutic responses and guided duration of therapy in critically ill patients.^5^ However, due to their unspecific character and insufficient predictive value for the individual, urgent identification of a more robust biomarker is essential.^6^

Systemic inflammation is related to alterations in circulating blood cell composition as a response of the immune system to stress or sepsis.^7^, ^8^ White blood cell (WBC) count has been identified as an important systemic inflammation marker.^9^ Recent reports demonstrated that the WBC count had an independent ability to predict all‐cause mortality.^10^, ^11^ Data from previous research indicated that changes in WBC (neutrophil and lymphocyte) were associated with inflammatory diseases. The most well‐known WBC disease is relative lymphopenia accompanied by neutrophilia. The neutrophil‐to‐lymphocyte ratio (NLR) is a rapid and simple parameter of systemic inflammation and stress, which expresses the severity of the disease in the critically ill patients.^8^ Lactate is a normal product of anaerobic metabolism.^12^ The blood lactate levels are elevated not only due to tissue hypoxia or anaerobic glycolysis, but also due to secondary activation of the stress response. Characteristics of lactate production best fit the notion of an adaptive survival response that grows in intensity as disease severity increases. Measurement of blood lactate levels may aid prediction of mortality. Its presence and progression are widely appreciated by doctors as an indicator in the detection of very high‐risk populations.^13^ Lactate measurement was frequently used in hospital settings to identify patients with sepsis and to guide initiation of early treatment.^14^

The primary purpose of this study was to determine the capability of NLR and plasma lactate to predict mortality during hospitalization and later (in 28 days) in patients with sepsis from the Department of Critical Care Medicine, compared to established sepsis‐related biomarkers (PCT and CRP).

## 2 METHODS

### 2.1 Patients

**Figure 1:**
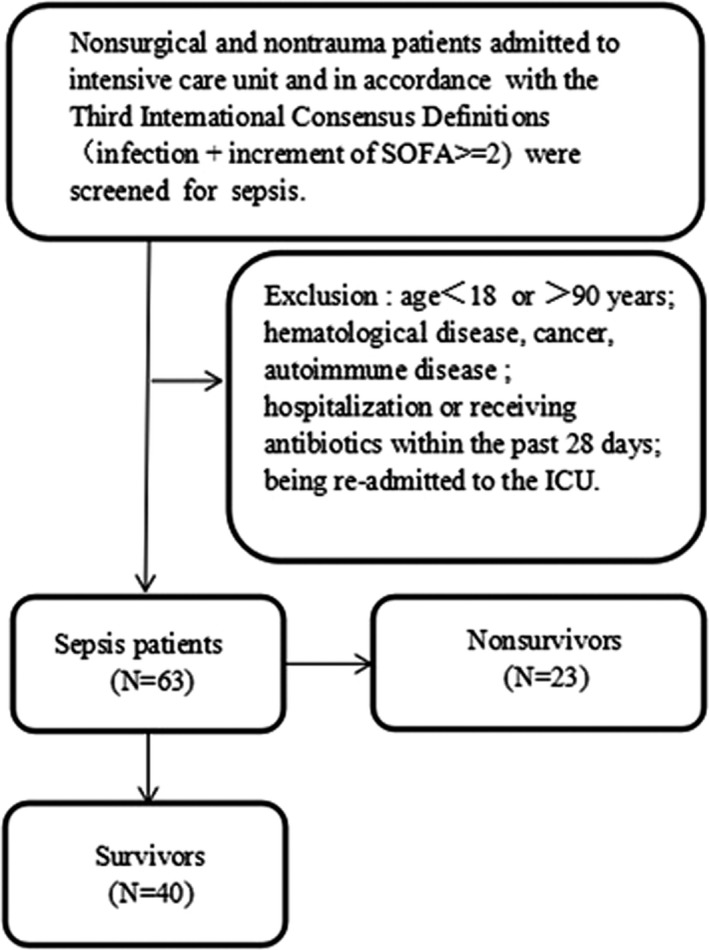
The algorithm of patient enrollment

We conducted a prospective, observational study at the Changhai Hospital, China, a 2000‐bed tertiary care hospital involving patients with sepsis. Sixty‐three nonsurgical and nontrauma adult patients with sepsis admitted to the Department of Critical Care Medicine from September 2016 to October 2018 were consecutively included in the study. Enrollment within 24 hours after onset of sepsis was chosen to eliminate confounding factors, such as variable onset time, and to improve group homogeneity. In the present study, sepsis and septic shock were defined in accordance with the Third International Consensus Definitions (infection + increment of SOFA ≥ 2).^15^ The exclusion criteria included the following: (a) age < 18 years or >90 years; (b) hematological disease, cancer, autoimmune disease; (c) hospitalization or receiving antibiotics within the past 28 days; and (d) being re‐admitted to the ICU. All eligible patients were divided into two groups: (a) the survivor group, which included patients who survived 28 days after initial diagnosis, and (b) the nonsurvivor group, which included patients who died within 28 days following diagnosis (Figure 1).

The protocol and procedure for obtaining informed consent were approved by the Institutional Ethical Committees, and consent forms were provided by first‐degree relatives or patients.

### 2.2 Measurements and data collection

Baseline characteristics of the patients including demographics (age and gender), comorbidities (cardiovascular disease, chronic lung disease, and diabetes, among others), and primary diagnosis were recorded. Blood samples were collected within 24 hours after diagnosis. Routine blood tests, blood gas analysis, and PCT concentrations were conducted in the hospital's central laboratory. Routine blood tests, including neutrophil count and lymphocyte count (blood analyzer, Sysmex XN9000), CRP (scattering nephelometry, reagents purchased from Shenzhen Guosai Biotechnology Co., Ltd.), and plasma PCT concentrations were measured in duplicates as recommended by the manufacturer using a time‐resolved amplified cryptate emission technology assay (PCT KRYPTOR; Brahms AG). Plasma lactate was evaluated on an ABL800 Flex (Radiometer Medical ApS). All samples were encrypted and analyzed by trained laboratory technicians blinded to the patients' diagnoses.

### 2.3 Statistical analysis

All analyses were performed using SPSS 21.0 software, and statistical significance was set at *P* < 0.05. Box plots were used to present results. The chi‐square test was used to compare the two sets of data, and the Mann‐Whitney *U* test was used to compare the two sets of measurement data. Receiver operating characteristic (ROC) curves were constructed to compare prognostic performance and to determine the optimal cutoff points for the biomarkers. Spearman's rank correlation coefficient (rs) was used to determine correlation between conventional inflammation‐related biomarkers.

## 3 RESULTS

### 3.1 Patient population and characteristics

Sixty‐three consecutive patients meeting the criteria of sepsis were enrolled, with 40 (63.5%) patients in the survivor group and 23 (36.5%) patients in the nonsurvivor group. Patients who underwent a rapid fatal outcome (within 24 hours after ICU admission) were excluded from the analysis. Baseline clinical characteristics of the 63 patients included in the present study are listed in Table 1.

**Table 1:** Baseline characteristics of patients enrolled in the study

	Survivors	Nonsurvivors	*P*‐value
n	40 (63.5%)	23 (36.5%)	
Age (mean ± SD; range)	66.7 ± 13.83	70.87 ± 12.07	0.781
Male gender	28 (70%)	17 (73.91%)	0.741
Body mass index (kg/m^2^)	20.97 ± 2.39	21.38 ± 3.11	0.123
Blood pressure (mm Hg)			
Systolic blood pressure	126.1 ± 29.25	116.39 ± 26.72	0.638
Diastolic blood pressure	73.6 ± 24.75	67.35 ± 18.37	0.645
Heart rate	93.92 ± 21.51	101.52 ± 26.21	0.234
Respiratory rate	21.1 ± 4.78	21.87 ± 4.8	0.437
Comorbidity	11 (27.5%)	7 (30.43%)	0.804
Cardiovascular disease	5 (12.5%)	6 (26.09%)	
Lung disease	2 (5%)	2 (8.7%)	
Diabetes	8 (20%)	3 (13.04%)	
Other	2 (5%)	4 (17.39%)	
Smoker	21 (52.5%)	14 (60.87%)	0.215
Alcohol	18 (45%)	12 (52.13%)	0.716
Site of infection			
Thorax only	3 (7.5%)	2 (8.7%)	
Abdomen only	14 (35%)	13 (56.52%)	
Urinary only	4 (10%)	1 (4.35%)	
Combined	2 (5%)	1 (4.35%)	
Other	17 (42.5%)	6 (26.09%)	
Organisms			
Gram‐positive isolates only	6 (15%)	4 (17.39%)	
Gram‐negative isolates only	12 (30%)	7 (30.43%)	
Fungi	1 (2.5%)	1 (4.38%)	
Mixed bacterial isolates	5 (12.5%)	4 (17.39%)	
Unidentified	16 (40%)	7 (30.43%)	
Dialysis	6 (15%)	6 (26.09%)	0.281
Mechanical ventilation	16 (40%)	14 (60.87%)	0.11
Vasopressors	12 (30%)	22 (95.65%)	＜0.01

Note

Data are presented as n (%), means (±SD), and medians (25th, 75th percentile).

Abbreviation: ICU, intensive care unit.

### 3.2 Levels of NLR, plasma lactate, PCT, and CRP

**Figure 2:**
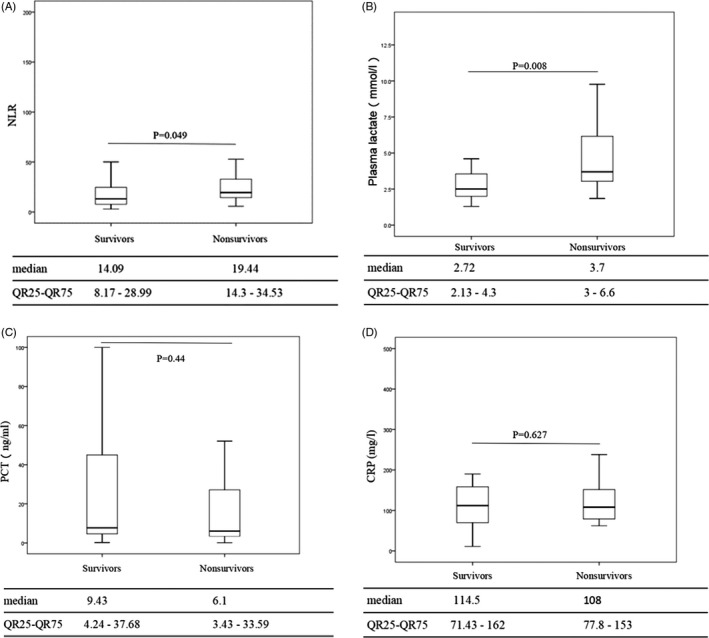
Levels of neutrophil‐to‐lymphocyte ratio (NLR), plasma lactate, procalcitonin (PCT), and C‐reactive protein (CRP)

Neutrophil‐to‐lymphocyte ratio and plasma lactate were higher in the nonsurvivors than in the survivors: Median (IQR25‐IQR75) values were 19.44 (14.33‐4.53) vs 14.09 (8.17‐28.99), *P* = 0.049; and 3.7 (3‐6.6) vs 2.72 (2.13‐4.3) ng/mL, *P* = 0.008, respectively. There were no statistical differences in the concentrations of PCT and CRP between nonsurvivors and survivors: 6.1 (3.43‐33.59) vs 9.43 (4.24‐37.68) ng/mL, *P* = 0.44; and 108 (77.8‐153) vs 114.5 (71.43‐162) ng/mL, *P* = 0.672, respectively (Figure 2).

### 3.3 Prediction of mortality

**Figure 3:**
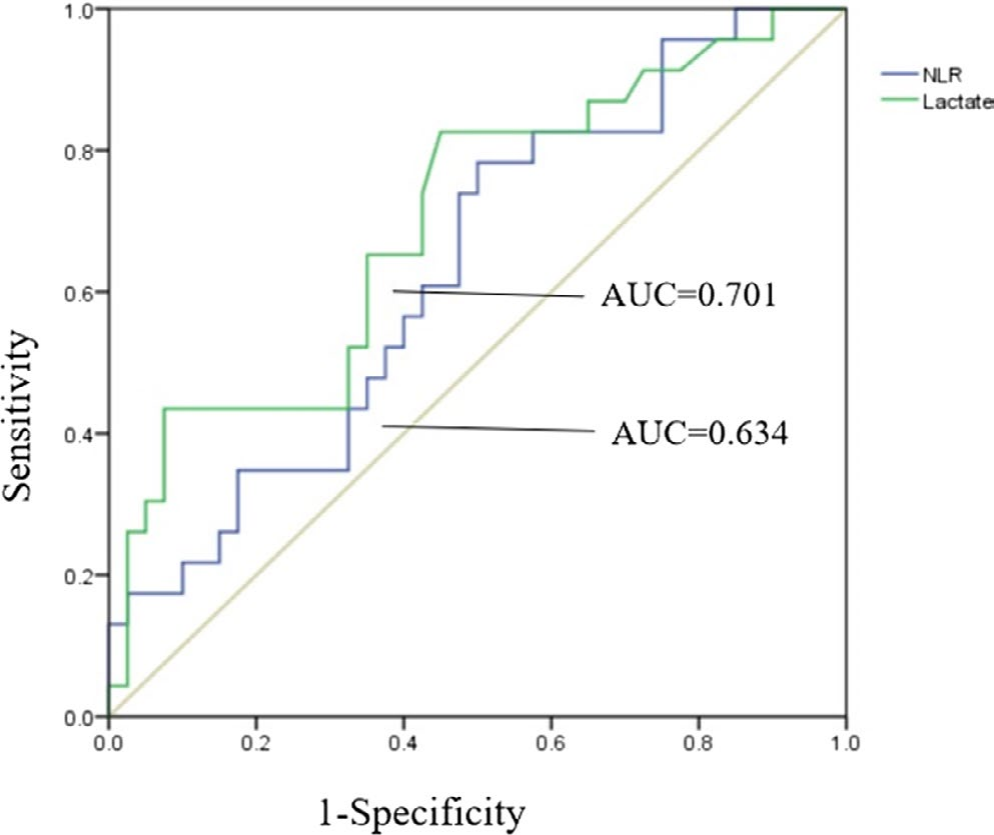
Receiver operating characteristics (ROC) of neutrophil‐to‐lymphocyte ratio (NLR) and plasma lactate levels for prediction of mortality in patients with sepsis. The area under the ROC curve was 0.634 for the ability of NLR level to predict 28‐d mortality, with a sensitivity of 78.3% and specificity of 50%. The area under the ROC curve was 0.701 for the ability of plasma lactate level to predict 28‐d mortality, and the sensitivity and specificity were 82.6% and 55%, respectively

**Figure 4:**
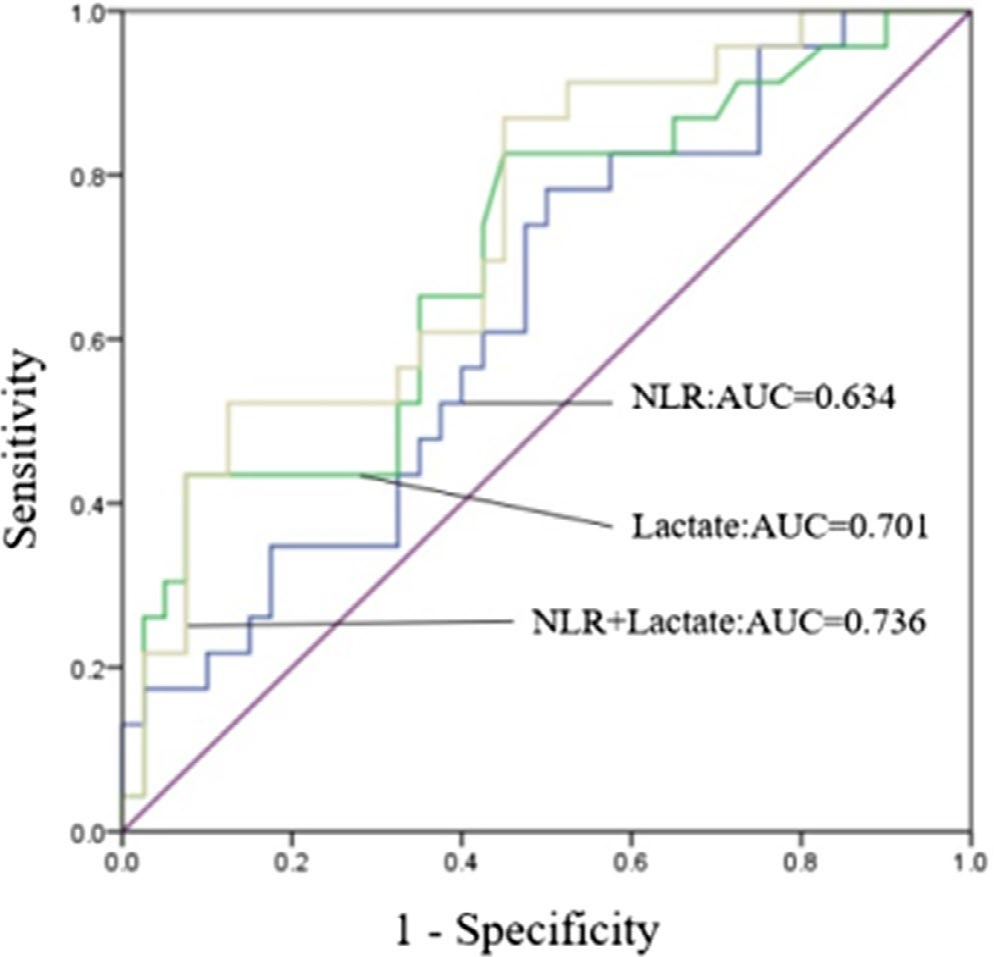
Receiver operating characteristics of neutrophil‐to‐lymphocyte ratio (NLR), lactate, and NLR with added lactate in patients with sepsis in relation to 28‐d mortality

Receiver operating characteristic curves were used to analyze the relationship between NLR, lactate, and 28‐day mortality. The area under the ROC curve was 0.634 (*P* = 0.049) for the ability of NLR to predict 28‐day mortality, with a sensitivity of 78.3% and specificity of 50%. The optimal cutoff was 14.08. The area under the ROC curve was 0.701 (*P* = 0.008) for the ability of plasma lactate level to predict 28‐day mortality, and the sensitivity and specificity, at an optimal cutoff value of 2.99 mmol/L, were 82.6% and 55%, respectively (Figure 3). Finally, further ROC curve analysis revealed that prediction of 28‐day mortality was improved markedly by addition of NLR to plasma lactate (AUC = 0.736, *P* = 0.002) compared to NLR (AUC = 0.634) and plasma lactate (AUC = 0.701) alone (Figure 4).

### 3.4 Correlation with conventional inflammation‐related biomarkers

Spearman's rank correlation coefficient (rs) was conducted to assess the correlations among NLR and lactate levels and biomarkers of inflammation (PCT and CRP). There was significant correlation between NLR and lactate (rs = 0.412, *P* = 0.001), between lactate and CRP (rs = 0.257, *P* = 0.042), and PCT (rs = 0.288, *P* = 0.022). However, there was no significant correlation between NLR and CRP (rs = 0.142, *P* = 0.268) and PCT (rs = 0.221, *P* = 0.082).

## 4 DISCUSSION

Differentiating nonsurvivors from survivors is an important issue in clinical practice. Biomarkers may be used to enhance diagnostic, prognostic, and theranostic values, all of which are important factors influencing the outcomes in patients with sepsis.^4^ Blood is a biological fluid traditionally used for biomarker studies. The current study evaluated the predictive value of four biomarkers—NLR, plasma lactate, PCT, and CRP.

It has already been known that levels of PCT and CRP may allow for a better outcome prediction. In fact, our findings did not indicate significant differences in the plasma concentrations of PCT and CRP between nonsurvivors and survivors. This result seems to substantiate those of a previous study involving patients critically ill with sepsis and infection, where nonsurvivors and survivors had the same PCT plasma levels.^5^ PCT levels have been shown to correlate with the severity of sepsis.^16^, ^17^ Furthermore, CRP levels of nonsurvivors did not differ significantly from those of survivors. This is consistent with other studies, demonstrating that CRP did not allow early discrimination of nonsurvivors from survivors with postoperative sepsis.^16^

Results of the current study indicated that NLR was significantly elevated in the nonsurvivors compared to that in the survivors. NLR may simply reflect an appropriate tissue perfusion response that is adaptive. There was significant correlation between NLR and lactate (rs = 0.412, *P* = 0.001). The association between increased mortality and raised NLR was also consistent with that in prior studies; Shahidi reported that the NLR was a simple, cheap, rapidly accessible, and independent indicator of short‐ and long‐term mortalities in critically ill patients.^18^ WBCs, in particular neutrophils and lymphocytes, are known to play an important role in the inflammatory processes of chronic diseases, such as atherosclerosis.^19^, ^20^ NLR also had proven useful for predicting intensive care suitability and mortality in patients with community‐acquired pneumonia.^21^ Neutrophils played an especially crucial role in the acute inflammatory response to tissue injury and were related to reperfusion injury.^22^ Lymphocytopenias were associated with cortisol production and neuroendocrine stress.^23^ NLR indicated that these two were inversely related immune pathways: one representing unrestrained inflammation and the other a latent immune pathway.^24^ Thus, the most plausible explanation was that NLR would be increased in nonsurvivors due to systemic inflammation. It had been proved that NLR may play an active role in stratifying risk, optimizing treatment, and managing patients.

Results of the present study suggested lactate as the single best‐performing biomarker for identifying nonsurvivors and survivors of sepsis. Under normal physiological conditions, the half‐life of lactate is approximately 20 minutes. Therefore, a persistently high lactate level reflected its continuous production or lack of elimination.^25^ Previous studies demonstrated that elevated lactate levels were associated with high mortality and poor prognostic outcomes.^26^, ^27^ Several biomarkers have been proposed to predict mortality, but lack sufficient sensitivity and specificity. Both composite biomarkers may have significantly higher sensitivity and specificity than all the single biomarkers, suggesting the need for a joint interpretation of several biomarkers in sepsis prediction.

In conclusion, raised NLR and lactate levels showed significant association with increased mortality in patients with sepsis. NLR and lactate may be important prognostic biomarkers to optimize treatment and manage patients. Larger, multicenter trials may be needed to further verify these findings.
